# P-2172. Real-World Use of Letermovir for Prophylaxis of Cytomegalovirus in High-Risk Kidney Transplant Recipients

**DOI:** 10.1093/ofid/ofaf695.2335

**Published:** 2026-01-11

**Authors:** Athena Matsikas, Srijana Jonchhe, Sapna A Mehta, fainareti Zervou

**Affiliations:** NYU Langone Health, New York, New York; NYU Langone Health, New York, New York; New York University Grossman School of Medicine, New York, NY; new york university, New york city, New York

## Abstract

**Background:**

Letermovir (LTM) is FDA approved for cytomegalovirus (CMV) prophylaxis (PPX) in high risk (D+/R-) kidney transplant recipients (KTR). Compared to valganciclovir (VGCV), LTM causes less myelosuppression and does not require renal dose adjustment. A dose of LTM costs over $300, creating operational challenges in LTM access. This study evaluates the efficacy of LTM in preventing CMV infection in D+/R- KTR, and highlights barriers to use in clinical practice.

**Figure 1:**
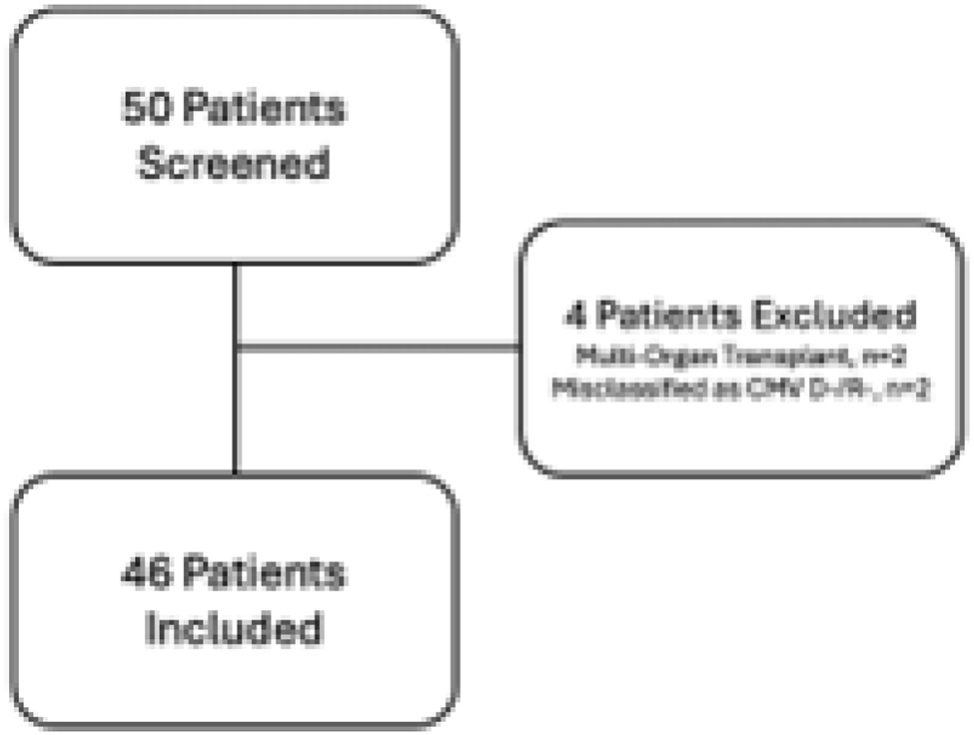
Study Population

**Table 1:**
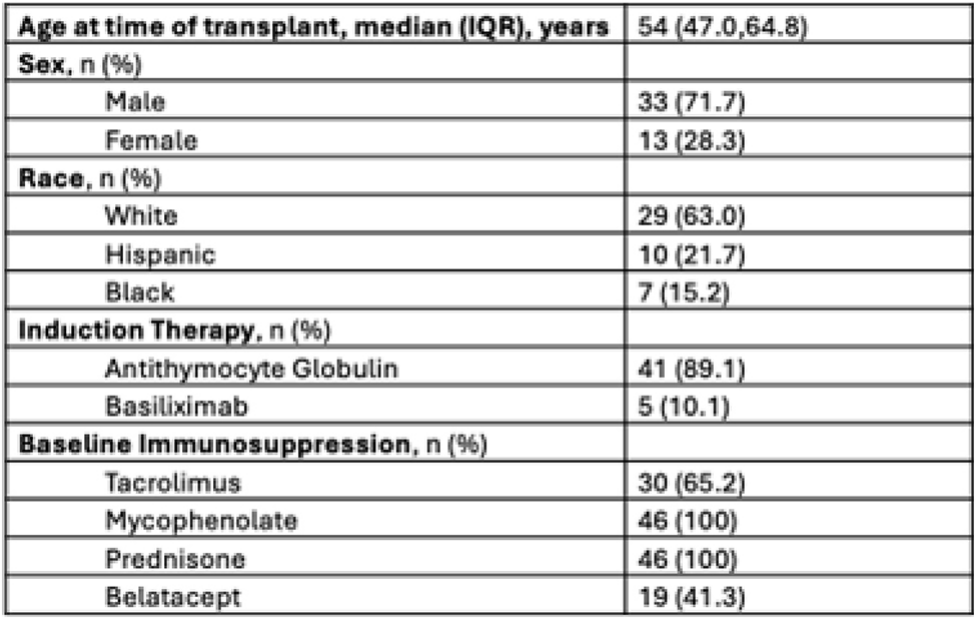
Baseline Characteristics

**Methods:**

Single-center retrospective chart review of all CMV D+/R- KTR transplanted between January 1, 2024 and October 31, 2024. The primary outcome was efficacy of LTM in preventing CMV viremia. Secondary outcomes include incidence of medication access barriers including prior authorization (PA) and financial assistance (FA), and description of LTM course.

**Table 2:**
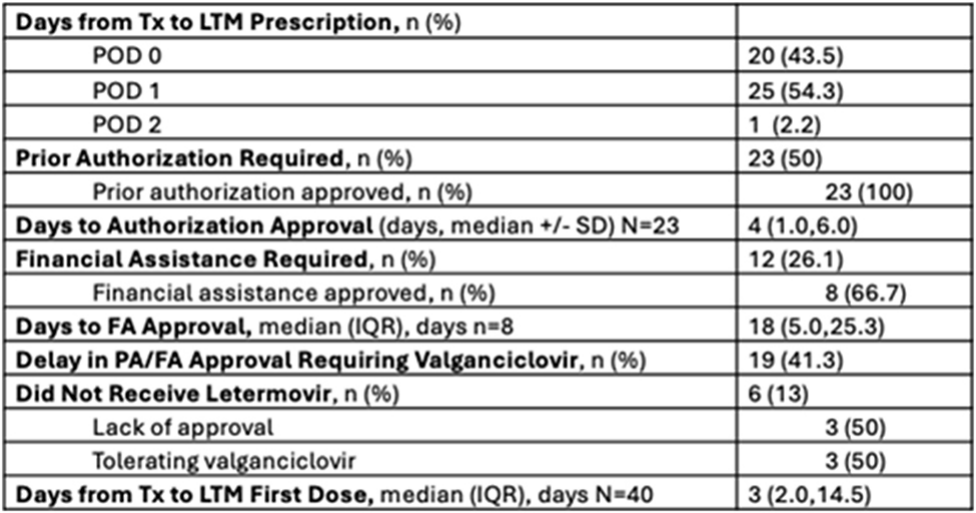
Letermovir Access

**Table 3:**
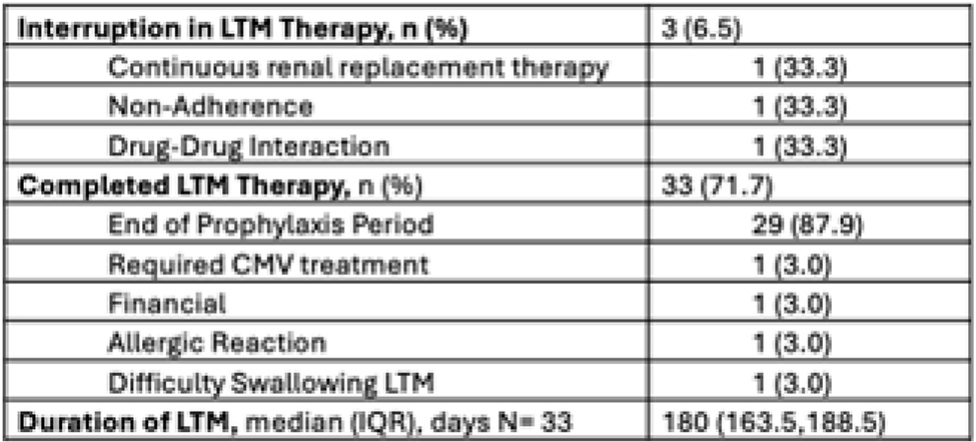
Letermovir Courses

**Results:**

Fifty CMV D+/R- kidney transplants were performed in the study interval. Four patients were excluded (Figure 1), leaving 46 patients in the analysis. Most KTR (89.1%) received antithymocyte globulin induction, and de-novo belatacept based immunosuppression was common (41.3%). LTM PPX was effective, with no breakthrough viremia in 45 KTR (97.8%). One KTR (2.2%) transitioned to CMV treatment for a viral load (VL) of 223 IU/mL in the setting of bacterial infection. Half the cohort (50%) required PA for LTM and 12 KTR (26.1%) needed FA due to high copay. Due to delay in accessing LTM beyond post-operative day 4, 19 KTR (41.3%) received valganciclovir (VGCV) while waiting for PA or FA. Six patients (13%) never started LTM. Details of access barriers are listed in table 2. At time of analysis, 33 KTR (71.7%) had stopped LTM. Premature discontinuation of LTM was uncommon, occurring in 4 KTR (12.1%), details of LTM courses are listed in table 3.

**Conclusion:**

LTM is an effective agent for CMV PPX in D+/R- KTR. However, LTM access remains challenging, with high PA and FA needs. These barriers required VGCV while access was pending. Broader data supporting the use of LTM for CMV PPX may help improve access and reduce operational barriers in the future.

**Disclosures:**

All Authors: No reported disclosures

